# Sustainable Graphene Synthesis and Analysis of Graphene-Based PLA Nanocomposites: Impacts of Polymer Functionalization and Potential Applications in Cancer Treatments

**DOI:** 10.1021/acsomega.5c01094

**Published:** 2025-06-05

**Authors:** Álefe Roger Silva França, Beatriz da Silva Batista, Joel Félix Silva Diniz Filho, Rosa Maria Viana Sousa, Alan Silva de Menezes, Clenilton Costa dos Santos, Ralph Santos-Oliveira, Pedro Filho Noronha Souza, Luzeli Moreira da Silva, Luciana Magalhães Rebêlo Alencar

**Affiliations:** † Center for Social Sciences, Health and Technology, 37892 Federal University of Maranhão, Advanced Unit, Imperatriz, MA 65900-410, Brazil; ‡ Department of Physics, Laboratory of Biophysics and Nanosystems, 37892 Federal University of Maranhão, Campus Bacanga, São Luís, MA 65080-805, Brazil; § Coordination of the Bachelor’s Degree In Natural Sciences − Physics/CCBA, Federal University of Maranhão, Campus Bacabal, Bacabal, MA 65700-000, Brazil; ∥ Laboratory of Nanoradiopharmacy and Synthesis of Novel Radiopharmaceuticals, Brazilian Nuclear Energy Commission, Nuclear Engineering Institute, Rio de Janeiro, Rio de Janeiro 21945-970, Brazil; ⊥ Laboratory of Radiopharmacy and Nanoradiopharmaceuticals, Rio de Janeiro State University, Rio de Janeiro 20550-013, Brazil; # Visiting Researcher at the Cearense Foundation to Support Scientific and Technological Development, Fortaleza, CE 60325-452, Brazil

## Abstract

Due to their optimized mechanical, physicochemical, and biocompatible properties, poly­(lactic acid) (PLA)-based composites functionalized with graphene have attracted growing interest in the biomedical field. In this study, a nanocomposite was developed by incorporating graphene at different concentrations into a commercial PLA-like resin, processed using the additive manufacturing technique of vat photopolymerization. Cell viability assays using human melanoma (MV3) tumor cells were performed on samples containing 0.1 and 0.3 wt % graphene. The results showed a significant, concentration-dependent inhibition of tumor cell proliferation. In contrast, nontumor cells (MNP-01 and MRC-5) exhibited good biocompatibility with the material, reinforcing the selective behavior of the composite. To better understand the composite’s anticancer potential and its interactions with cells, extensive structural, topographical, and vibrational characterizations were conducted. X-ray diffraction (XRD) analysis confirmed that the addition of graphene did not significantly alter the polymer’s structural profile. Raman spectroscopy yielded similar findings, showing unchanged vibrational modes. Atomic force microscopy (AFM) revealed changes in surface roughness, while Vickers microhardness (VM) tests showed increased hardness with higher graphene content. Wettability tests supported the biological findings, indicating increased hydrophilicity with higher graphene concentrations, which correlates with decreased MV3 cell viability. These results help elucidate the mechanisms mediating the interaction between cells and the PLA/graphene composite surface.

## Graphical abstract

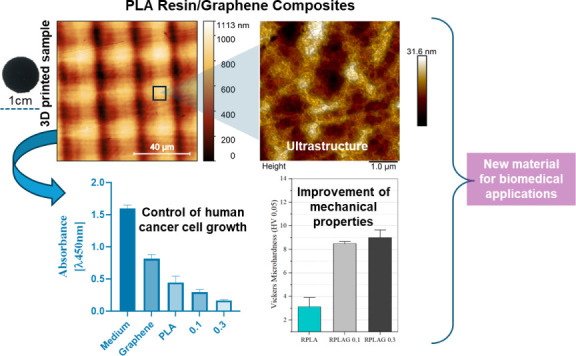

## 1 Introduction

Since it was first isolated in 2004,^1^ graphene has emerged as an exceptionally versatile material, with applications spanning various fields, including regenerative medicine,^2^ electronics and optoelectronics,^3^ energy storage,^4^ sensors,^5^ water filtration,^6^ and in the production of nanocomposites.^7^ Graphene’s widespread relevance stems from its exceptional properties, including large surface area, outstanding mechanical strength, excellent thermal and electrical conductivity, and remarkable optical characteristics.^8–12^ These features are largely attributed to the hexagonal lattice arrangement of carbon atoms and the two-dimensional structure, which allows for the confinement of electrons in two dimensions.^13,14^

According to Ghosal, Krishanu, and Kishor, Sarkar,^15^ graphene and its derivatives, such as graphene quantum dots (GQDs), graphene oxide (GO), and reduced graphene oxide (rGO), have been extensively explored in biomedical applications. These materials have been successfully used in biosensors,^16^ in tissue engineering,^17^ and drug delivery systems.^18^ Despite significant advances, challenges remain in the synthesis of graphene with high structural quality via environmentally friendly (“green”) methods and in the scalability of such processes, especially in light of increasing global demand.

Compared to traditional materials used in industrial applications (e.g., metals, glass, and petroleum-based plastics), composites offer several advantages, including reduced weight, enhanced mechanical and thermal performance, and corrosion resistance.^19,20^ From a biomedical standpoint, composites offer improved biocompatibility, enhanced cell adhesion, controlled degradation, and antimicrobial and anticancer potential.^21,22^ Polymeric composites, in particular, are attractive due to their lightweight nature, specific strength, acid and alkali resistance, and ease of processing.^23^

As highlighted by Andoh, Vivian et al.,^24^ nanocomposites (defined as materials in which at least one phase is in the nanoscale—whether carbon-based, polymeric, metallic, lipidic, ceramic, or hybrid) have opened new avenues for cancer diagnosis and therapy. These nanostructured materials (e.g., graphene derivatives, metallic nanoparticles, and quantum dots) offer unique physicochemical properties that enhance both detection and therapeutic effectiveness.

Driven by growing concerns about plastic waste and environmental sustainability,^25^ the global scientific community has sought alternatives such as biodegradable and biocompatible polymers. These materials have been explored in sustainable packaging,^26^ cosmetics,^27^ and textiles.^28^ Among them, poly­(lactic acid) (PLA) has gained prominence due to its biodegradability, biocompatibility, and derivation from renewable resources (e.g., corn, wheat). PLA is widely applied in scaffolds,^29^ medical devices and implants,^30^ and drug delivery systems.^31^

Farah et al.^32^ emphasized PLA’s highly tunable properties, including density, heat capacity, rheological and mechanical behavior, solubility, and degradation profile, as key to its broad application and commercial success. Importantly, these properties are influenced by the degree of crystallinity, which depends on its molecular configuration (D/L-PLA, PDLA, PLLA) and processing conditions. For instance, PLLA, which is highly crystalline, exhibits greater stiffness and elastic modulus compared to the amorphous PDLA.

Adekoya et al.^33^ noted that PLA/graphene composites have been extensively studied due to the synergy between these components, resulting in superior performance and making them excellent candidates for biomedical applications, such as drug delivery, tissue engineering, sensing, and anticancer therapies. Despite these advances, further efforts are needed to optimize their synthesis and processing and better understand their biological interactions.

Additive manufacturing (AM), also known as 3D printing, has emerged as a powerful tool for fabricating devices and prototypes with high precision, reduced time, and cost-efficiency.^34^ Different AM techniques are available depending on the materials used (filaments, powders, resins) and the fabrication mechanisms (e.g., extrusion, photopolymerization, laser sintering), each with varying resolution and surface finish degrees. However, as Quan et al.^35^ pointed out, photosensitive resins commonly used in photocuring AM methods, such as stereolithography (SLA) and digital light processing (DLP), often suffer from cytotoxicity, limiting their use in biomedical applications.

To overcome this limitation, the development of new biocompatible resin composites is crucial to expand the use of SLA and similar technologies in biological contexts. In this study, a PLA-like resin functionalized with multilayer graphene was developed and processed via SLA 3D printing, aiming at applications in cancer treatment. The composite was thoroughly characterized by XRD, Raman spectroscopy, AFM, SEM, microhardness testing, wettability analysis, and cell culture assays. Additionally, a green synthesis method was employed to obtain graphene, in line with the current trend toward sustainability. Further synthesis details are provided in the Supporting Information.

## 2 Materials and Methods

### 2.1 Graphene Synthesis

Graphene was synthesized via electrochemical exfoliation using commercial pencil graphite (grade 8B). This material’s composition (graphite combined with clay) facilitates the exfoliation process, as the nonexfoliated graphite and residual clay tend to settle at the bottom of the container.^36^ The exfoliation was carried out in an aqueous sodium sulfate solution (10.65 g of Na_2_SO_4_ in 75 mL of distilled water). A graphite rod connected to the positive terminal of a power source served as the anode, while a platinum wire connected to the negative terminal served as the cathode. A constant voltage of 10 V was applied for 15 min, or until complete exfoliation of the graphite was achieved. Subsequently, the exfoliated material underwent mechanical separation of the graphene layers via tip sonication at 40 kHz for 30 min. The resulting dispersion was purified through a washing step involving centrifugation at 4000 rpm for 30 min. The sedimented material was retained, and the supernatant was discarded. This process was repeated four times with the addition of distilled water at each cycle. The resulting graphene was dried at 90 °C for 30 min. The quality and structure of the material were evaluated using X-ray diffraction (XRD) and Raman spectroscopy (RS), as presented in the Supporting Information.

### 2.2 Preparation of PLA/Graphene Resin

To prepare the graphene-functionalized bioresin, graphene synthesized as described in Section 2.1 was added to a commercial PLA-like bioresin (Esun). In a beaker, 250 mL of resin was mixed with graphene at concentrations of 0.1 and 0.3 wt %. The mixture was then homogenized by tip sonication at 80% amplitude (40 kHz) for 30 min. After sonication, the resulting composite was used for sample printing. The selected graphene concentrations were based on previously reported biocompatibility and cytotoxicity thresholds, which can vary depending on cell type and experimental conditions.^37–39^

### 2.3 Sample Printing

The initial step in printing the samples involved design using CAD software, specifically Autodesk Fusion 360. Circular discs measuring 10 mm in diameter and 2 mm in thickness were modeled. This geometry was selected to match the dimensions of the wells in standard cell culture plates and the sample holder used in atomic force microscopy (AFM). The resulting models were exported to the Halot Box slicing software, which is freely provided by the printer manufacturer. In this step, key printing parameters were configured, including exposure times for the initial and subsequent layers, layer thickness, and motor speed. After configuration, the sliced files were exported and transferred to the printer. The samples were printed using a Creality Halot Mage 8K stereolithography (SLA) 3D printer.

### 2.4 X-ray Diffraction (XRD)

The samples’ diffractograms were obtained from a Bruker diffractometer, model D8 Advance, equipped with Cu-Kα radiation (λ = 1.5406 Å), a voltage of 40 kV, a current of 40 mA, and a linear detector. The data were obtained in the 2θ range from 5° to 75°, in an angular step of 0.02°, counting time 2 s per step.

### 2.5 Raman Spectroscopy (RS)

Raman spectroscopy was performed using a Horiba/Jobin-Yvon T64000 spectrometer, configured to achieve a spectral resolution of 0.02 cm^–1^. A green laser (λ = 532 nm, ∼ 14 mW at the sample surface) was used as the excitation source. The scattered signal was detected using a charge-coupled device (CCD) detector cooled with liquid nitrogen. The laser was focused onto the sample surface through a BX41 Olympus microscope equipped with a 100× objective, which also directed the scattered light to the spectrometer. Each spectrum was obtained from five consecutive acquisitions, each with an integration time of 15 s.

### 2.6 Scanning Electron Microscopy (SEM) and Energy-Dispersive X-ray Spectroscopy (EDS)

SEM analyses were carried out using EVO 15 equipment from Zeiss, using a variable pressure SE detector (VPSE) at variable pressure mode. Information about the chemical composition of the samples was obtained using Bruker’s EDS XFlash 410 detector.

### 2.7 Atomic Force Microscopy (AFM)

The Atomic force microscopy (AFM) measurements were performed in Quantitative Nanomechanical Mapping (QNM) mode using a Bruker MultiMode 8 scanning probe microscope equipped with a NanoScope V controller. Silicon probes with a nominal spring constant of 0.5 N/m and a tip radius of less than 10 nm were employed. All measurements were carried out at room temperature under controlled humidity conditions. The scan rate was set to 0.5 Hz, and the force–distance curves were acquired at a frequency of 1 kHz.

### 2.8 Vickers Microhardness (MV)

The samples’ MV values were obtained from a Vickers HMV-2T microhardness tester from Shimadzu. Ten (10) indentations were produced on the surface of each sample, applying a load of 0.49 N (or ≈ 0.05 kgf) for 15 s.

### 2.9 Wettability Tests

Wettability tests were conducted on RPLA, RPLAG 0.1 wt %, and RPLAG 0.3 wt % samples using distilled water. The contact angle was measured using the Young–Laplace method^40^ via the DropSnake plugin in the ImageJ software. This tool allows droplet shape fitting by manually placing points along the liquid–solid interface, followed by the generation of tangents at the contact points, from which the contact angle is calculated based on their average values (Figure 1A).

**Figure 1:**
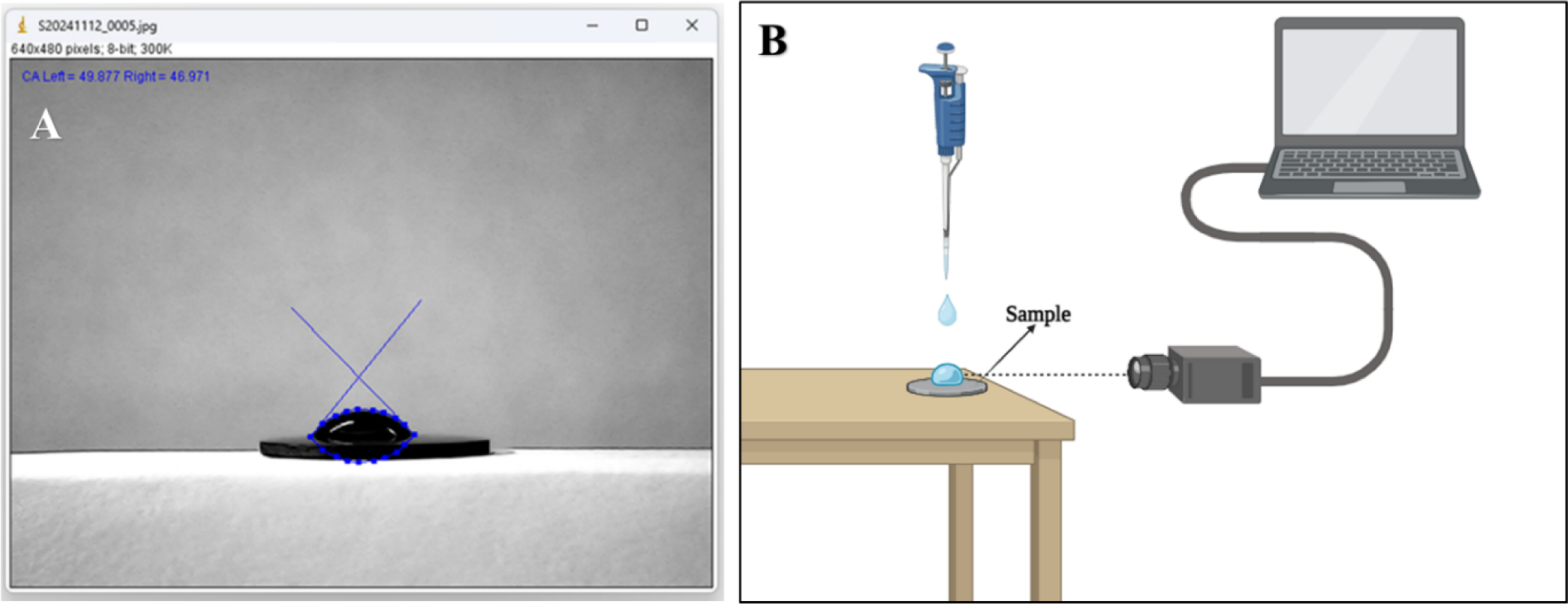
**ImageJ interface and experimental setup for wettability analysis.** A. Manual delimitation of the droplet contour using blue points in the DropSnake tool, with tangent lines fitted at the contact points to calculate the contact angle. B. Schematic representation of the experimental apparatus. Samples were placed on a flat surface, and 5 μL of distilled water was deposited using a pipet. A camera positioned parallel to the sample surface captured side-view images at 20-s intervals to determine the contact angle.

Figure 1B illustrates the experimental apparatus used for the contact angle measurements. A digital microscope was positioned at the same height as the sample platform to eliminate parallax effects. A 5 μL droplet of distilled water was then deposited onto the sample surface using a pipet held at a 90° angle. Side-view images were captured at 20-s intervals over a 3 min period. Five measurements were performed for each sample, and between measurements, the samples were washed with isopropyl alcohol and cleaned in an ultrasonic bath for 10 min to ensure surface consistency.

### 2.10 Cell Viability Assay

Cell viability assays were conducted using pure PLA resin and PLA-based samples containing 0.1 and 0.3 wt % graphene. The experiments began 24 h after seeding the cells in culture plates. The adhesion and viability of melanoma cells (MV3 cell line, obtained from the Rio de Janeiro Cell Bank^41^ were evaluated to assess the material’s potential to inhibit tumor cell growth. Additionally, the same samples were tested against nontumor cell lines, MNP-01 (nonmalignant gastric cells) and MRC-5 (fibroblasts), to investigate cytotoxicity. After 24 h of treatment, MTT solution (3-[4,5-dimethylthiazol-2-yl]-2,5-diphenyltetrazolium bromide) was added at a final concentration of 1 mg/mL in a total volume of 200 μL per well. Viable cells metabolize MTT into water-insoluble formazan crystals, which were subsequently dissolved in 200 μL of dimethyl sulfoxide (DMSO). Absorbance was measured at 450 nm using a microplate reader to quantify cell viability.^42^

## 3 Results

The graphene synthesized by the electrochemical route described in Section 2.1 was characterized by X-ray diffraction (XRD) and Raman spectroscopy. The corresponding results are presented in the Supporting Information. This graphene was incorporated into the PLA-like resin (RPLA) at concentrations of 0.1 wt % (RPLAG 0.1) and 0.3 wt % (RPLAG 0.3). Figure 2 displays the 3D-printed samples, illustrating the RPLA’s transparency and the gradual increase in opacity with higher graphene content in the polymer matrix.

**Figure 2:**
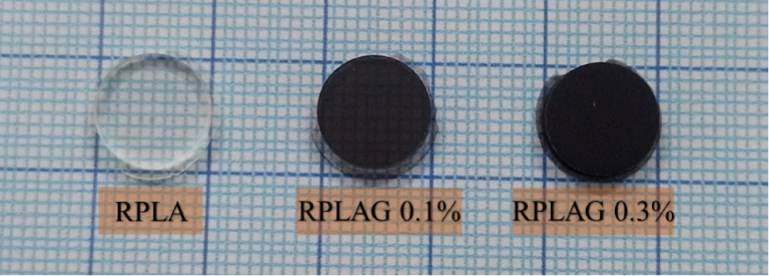
**PLA resin samples**. Samples printed from pure PLA resin (RPLA) and functionalized with 0.1 and 0.3 wt % graphene (RPLAG 0.1 and RPLAG 0.3).

Figure 3 shows the X-ray diffraction (XRD) patterns of the RPLA, RPLAG 0.1, and RPLAG 0.3 samples. All samples exhibit similar diffractograms, characterized by a broad amorphous halo spanning approximately 2θ ≈ 10° and ≈ 27°, which reflects the predominantly amorphous nature of the resin.

**Figure 3:**
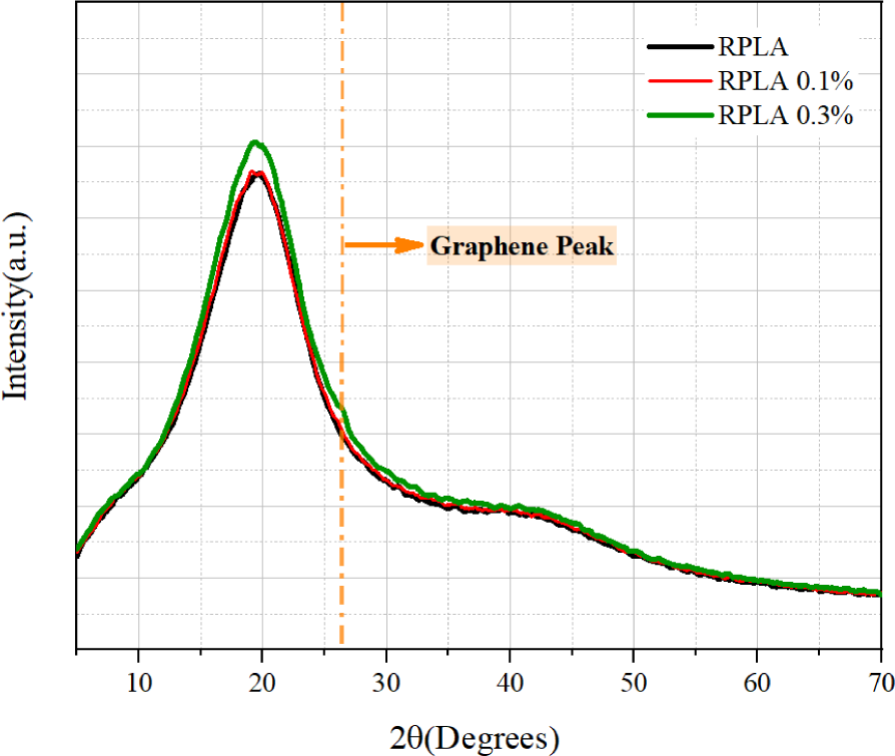
**Diffractogram of the RPLA and RPLAG 0.1–0.3 samples.** The data shows that the samples have an amorphous profile.

Figure 4 presents the Raman spectra of the RPLA, RPLAG 0.1 wt %, and RPLAG 0.3 wt % samples. Table 1 summarizes the characteristic vibrational modes of organic compounds identified in the spectra. All samples exhibited the same vibrational bands, differing only in the intensity of specific peaks, such as the symmetric stretching of C**–**O**–**C (υ_s_) and the symmetric bending of CH_3_ groups (δ_s_).^43^

**Figure 4:**
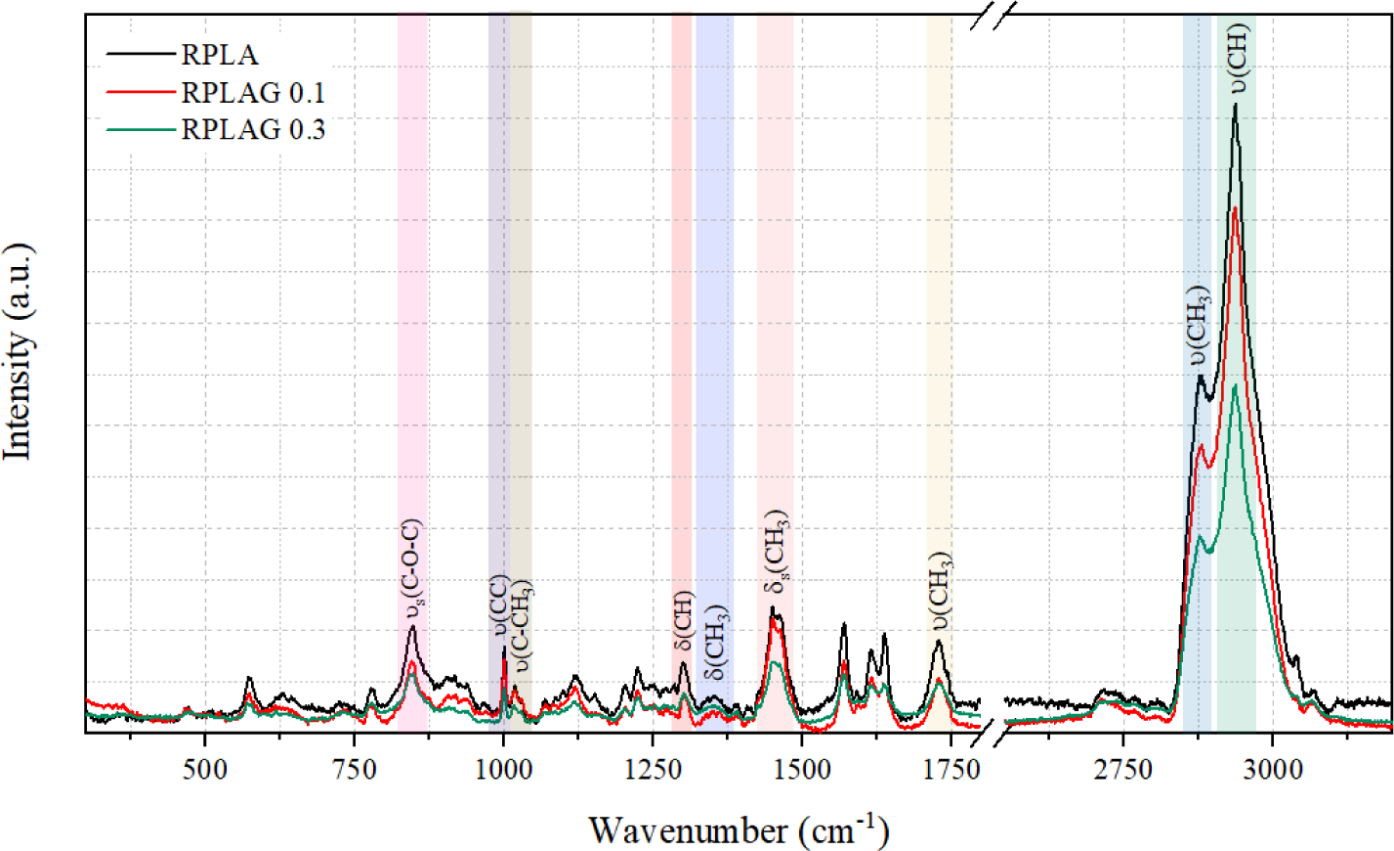
Raman spectrum of the RPLA and RPLAG 0.1–0.3 samples.

**Table 1:** Characteristic Vibrational Modes Observed for RPLA and RPLAG 0.1–0.3 Samples

Vibrational Signature	Wavenumber (cm^–1^)
υ(CH)	2935
υ(CH_3_)	2880
υ(C=O)	1726
δ_s_(CH_3_)	1460
δ(CH_3_)	1350
δ(CH)	1300
υ(C–CH_3_)	1020
υ(CC)	1000
υ_s_(C–O–C)	850

Figure 5 displays the SEM/EDS data for the RPLA, RPLAG 0.1 wt %, and RPLAG 0.3 wt % samples. The SEM micrographs (Figure 5a–c) reveal surface features composed of the same material as the polymer matrix, as confirmed by the corresponding elemental maps (Figure 5d–l), which show a uniform distribution of the detected elements. The EDS spectra (Figure 5m–o) confirm the presence of carbon (C), the primary constituent of the organic molecular structure of the samples. Additional elements detected, such as aluminum (Al) and calcium (Ca), are attributed to the intrinsic composition of the resin, as their uniform distribution across the matrix is evident in the elemental maps.

**Figure 5:**
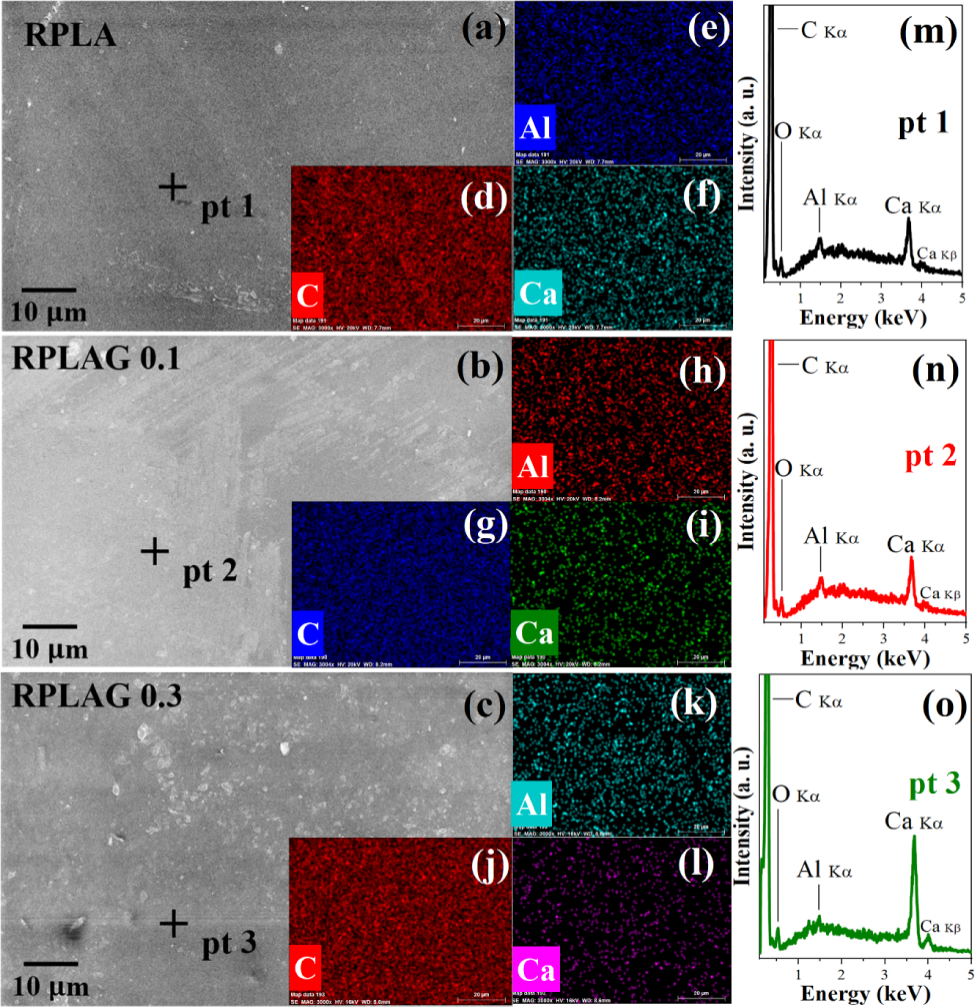
**SEM/EDS data for the RPLA and RPLAG 0.1–0.3 samples**. In (a–c), the photomicrograph of the sample surfaces and the respective points 1, 2, and 3 at which the EDS (m–o) measurements were carried out can be seen. The respective compositional maps of the carbon (C), aluminum (Al), and calcium (Ca) elements present in the samples (d–l) are presented.

Figure 6 presents the 100 × 100 μm^2^ topographic maps and corresponding line profiles, highlighted by dotted lines, for the RPLA, RPLAG 0.1 wt %, and RPLAG 0.3 wt % samples. All samples exhibit surface ripples resembling a grid-like pattern. The incorporation of graphene into the resin appears to modify the morphology of these surface features. Line profile analysis reveals that the average lateral spacing between surface depressions (valleys) remains approximately constant across all samples, with a mean distance of ∼ 27 μm. However, the vertical distance between peaks and valleys (h) decreases as the graphene content increases in the resin matrix, with amplitudes of approximately 510 nm for RPLA, 480 nm for RPLAG 0.1, and 350 nm for RPLAG 0.3.

**Figure 6:**
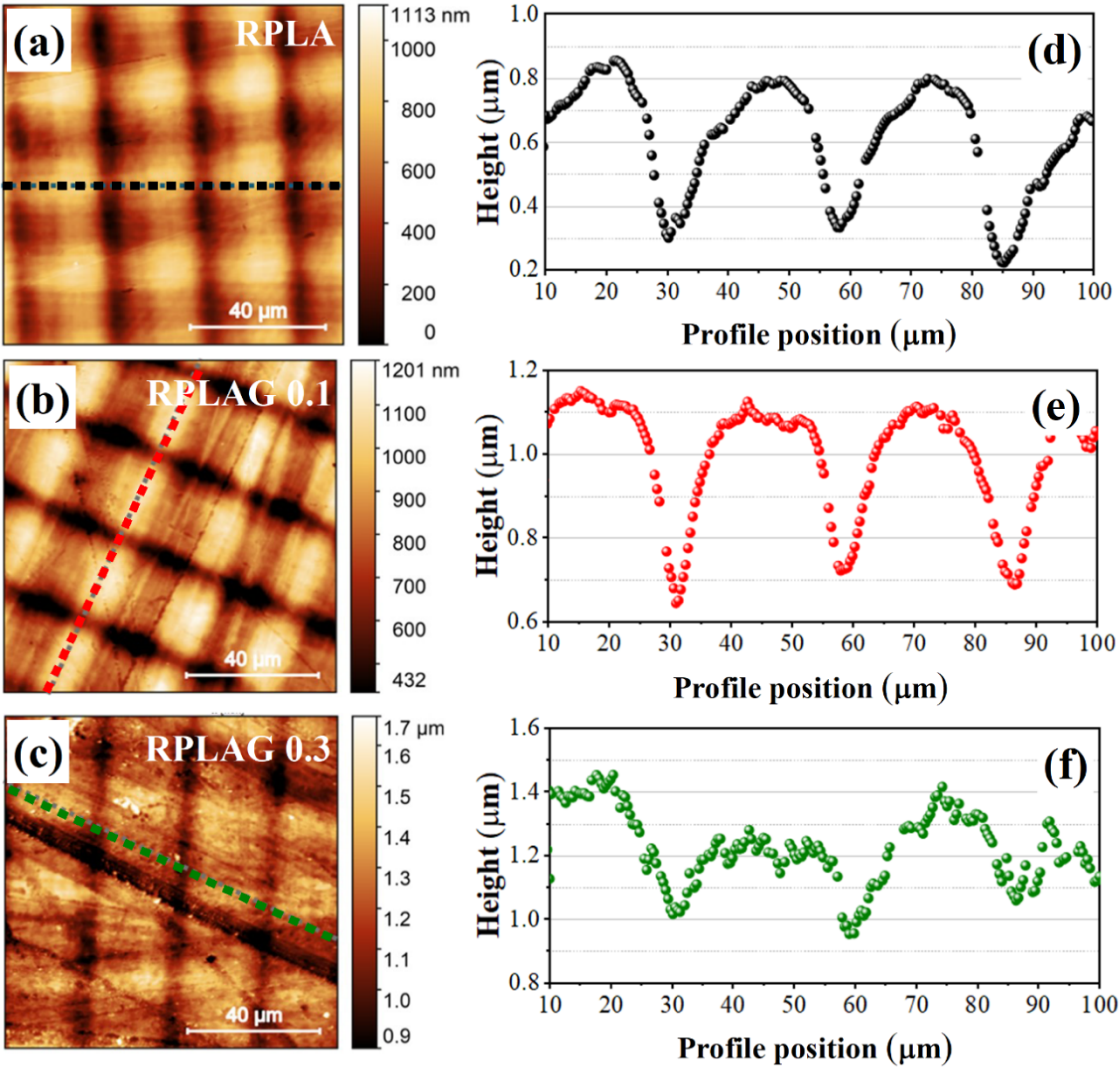
**Topographic information on RPLA and RPLAG 0.1–0.3 samples from 100 × 100 μm scan maps.** In (a–c), one can observe the topographic maps of the sample surfaces and the respective profiles of the dotted lines presented in (d–f).

To better assess the morphological changes induced by graphene incorporation into the PLA matrix, 5 × 5 μm^2^ surface maps were acquired using atomic force microscopy (AFM) in Quantitative Nanomechanical Mapping (QNM) mode. Figure 7 presents high-resolution topographic images of the sample surfaces, revealing the presence of pores, particularly in the RPLAG 0.2 sample. Variations in root-mean-square roughness (Rq) were observed among the samples; however, no linear correlation was found between Rq and graphene concentration. From the three-dimensional (3D) perspective shown in Figure 7d–f, the surface topography appears progressively more homogeneous with increasing graphene content.

**Figure 7:**
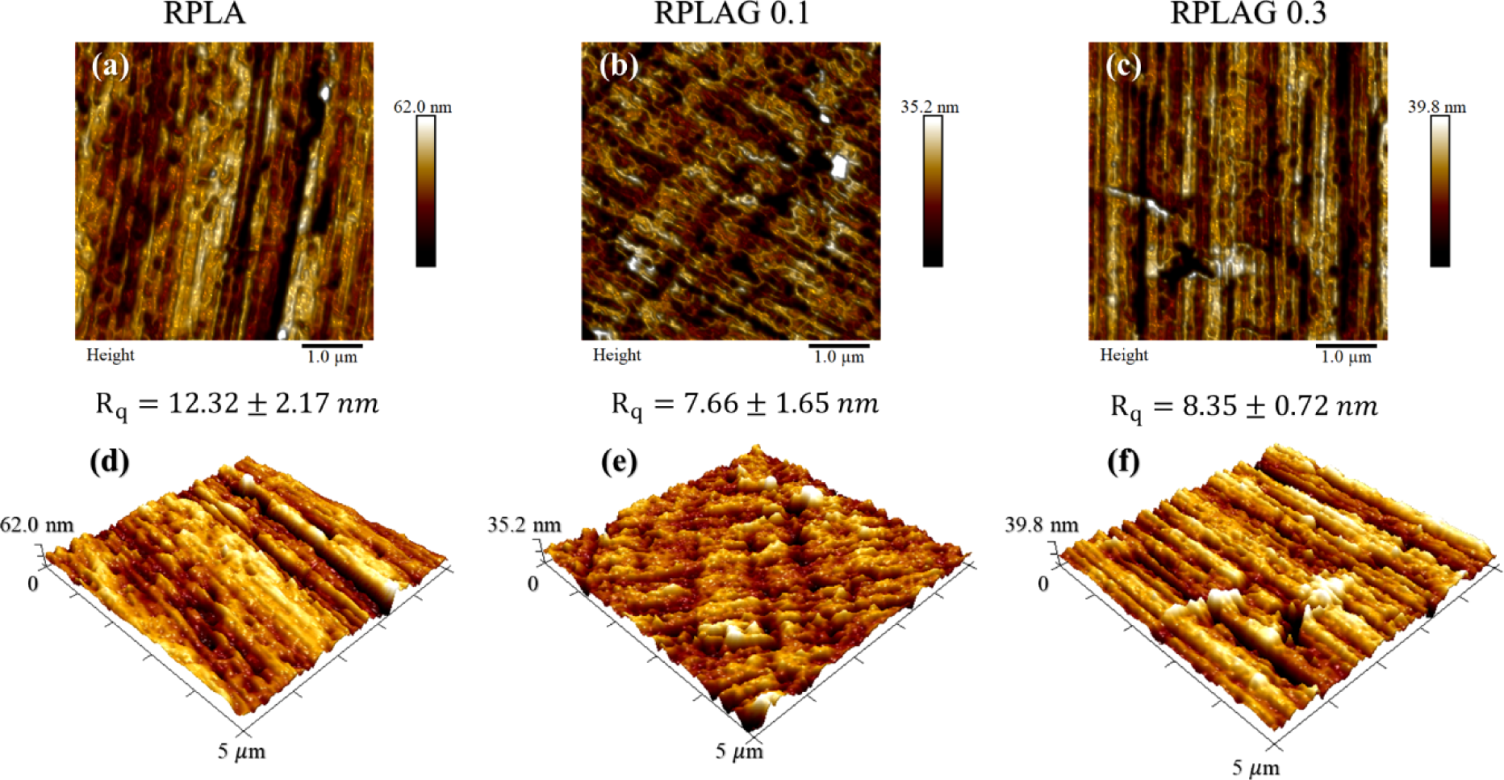
**Topographic information on RPLA and RPLAG 0.1–0.3 samples from 5 × 5** **μm scan maps.** In (a–c), the topographic maps of the sample surfaces in two dimensions are observed, and the respective three-dimensional maps in (d–f) are also observed.

Figure 8 shows the comparative Vickers microhardness (MV) values for the RPLA, RPLAG 0.1 wt %, and RPLAG 0.3 wt % samples. The incorporation of graphene into the polymer matrix led to a noticeable increase in local microhardness. For the MV measurements, a load of 0.05 kgf was applied. At 0.1 wt % graphene, the composite exhibited a marked increase in hardness, rising from 3.0 ± 1.0 HV (pure RPLA) to 8.5 ± 0.2 HV.

**Figure 8:**
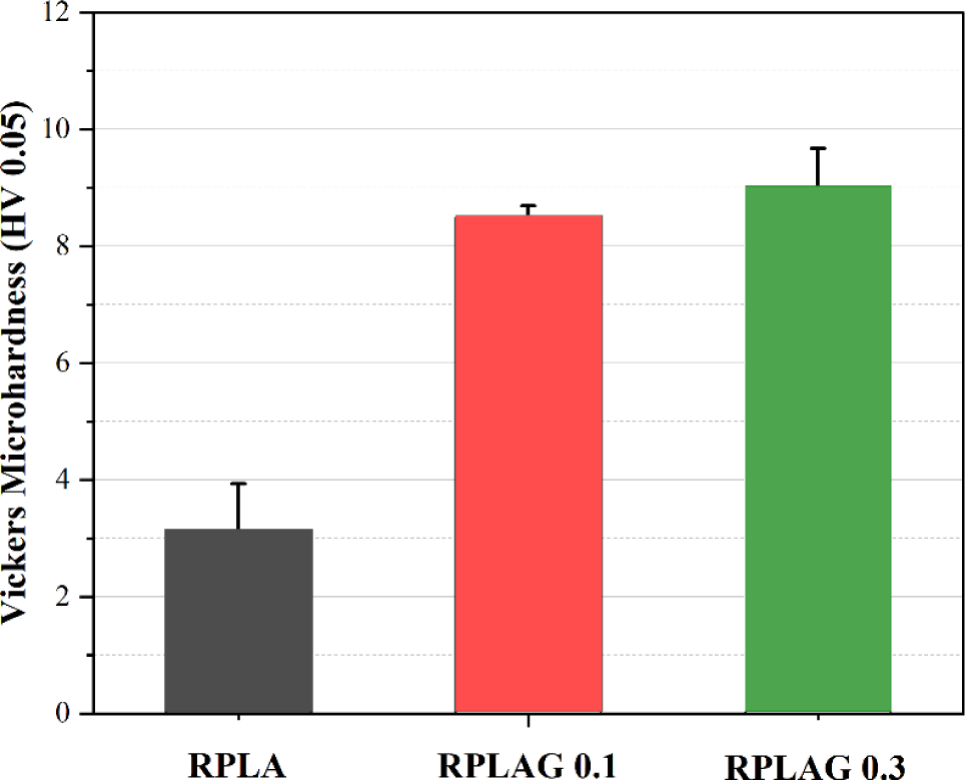
**Vickers microhardness of RPLA and RPLAG 0.1–0.3 samples**. The average MV values of the RPLA, RPLAG 0.1, and RPLAG 0.3 samples are 3.0 ± 1.0 HV, 8.5 ± 0.2 HV, and 9.0 ± 0.7HV.

Wettability tests were performed to investigate the influence of multilayer graphene incorporation on the hydrophobic/hydrophilic behavior of PLA. Figure 9 shows the variation in the contact angle of a distilled water droplet deposited on the surface of RPLA, RPLAG 0.1 wt %, and RPLAG 0.3 wt % samples over time (in seconds). As shown in Figure 9A, the contact angle decreases with increasing graphene content, indicating enhanced wettability as a result of a substantial increase in the hydrophilicity of the PLA/graphene nanocomposites.^44^ Representative images of the water droplet on the sample surfaces at the initial time point (*t* = 0 s) are shown in Figure 9B–D, and at the final time point (*t* = 180 s) in Figure 9E–G. Based on the slope of the fitted curves (Figure 9A), the spreading rates of the RPLA and RPLAG 0.1 samples were found to be similar, while the RPLAG 0.3 sample exhibited a slight decrease in spreading rate, suggesting a marginal reduction in droplet mobility on the surface.

**Figure 9:**
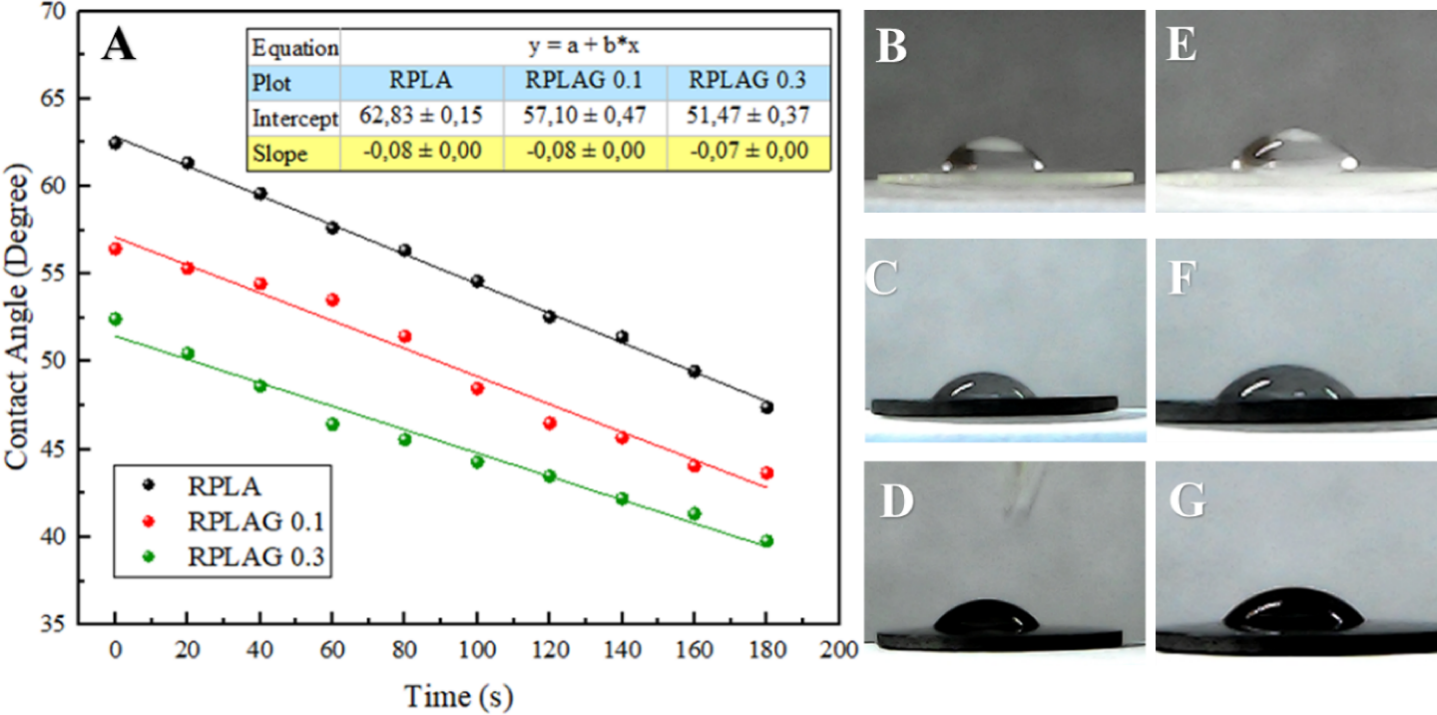
Wettability analysis of RPLA, RPLAG 0.1 wt %, and RPLAG 0.3 wt % samples. A. Variation in contact angle of a distilled water droplet over time for each sample. The slope of the linear fit indicates similar spreading rates across the samples. B–D. Initial droplet shape on the surfaces of RPLA, RPLAG 0.1, and RPLAG 0.3, respectively (*t* = 0 s). E–G. Droplet spreading after 180 s on the same samples.

Cell viability assays were conducted to evaluate the interaction between cancer cells and the surfaces of RPLA, RPLAG 0.1 wt %, and RPLAG 0.3 wt % samples (Figure 10A). The RPLA sample and pure graphene both supported some degree of viability for the MV3 melanoma cell line. However, when graphene was incorporated into the PLA matrix, a significant, dose-dependent reduction in cell viability was observed with increasing graphene concentration. An inverse relationship was therefore identified between MV3 cell viability and graphene content in the composite. Regarding cytotoxicity against nontumor cells, the results were even more favorable. While pure graphene exhibited pronounced cytotoxicity (Figure 10B), the PLA/graphene composites at both 0.1 and 0.3 wt % concentrations showed substantially lower toxicity toward noncancerous cell lines. For MNP-01 cells, viability remained at 95%, 89%, and 75% for RPLA, RPLAG 0.1, and RPLAG 0.3, respectively. In the case of MRC-5 fibroblasts, viability was 92%, 95%, and 89% for the same samples (Figure 10B).

**Figure 10:**
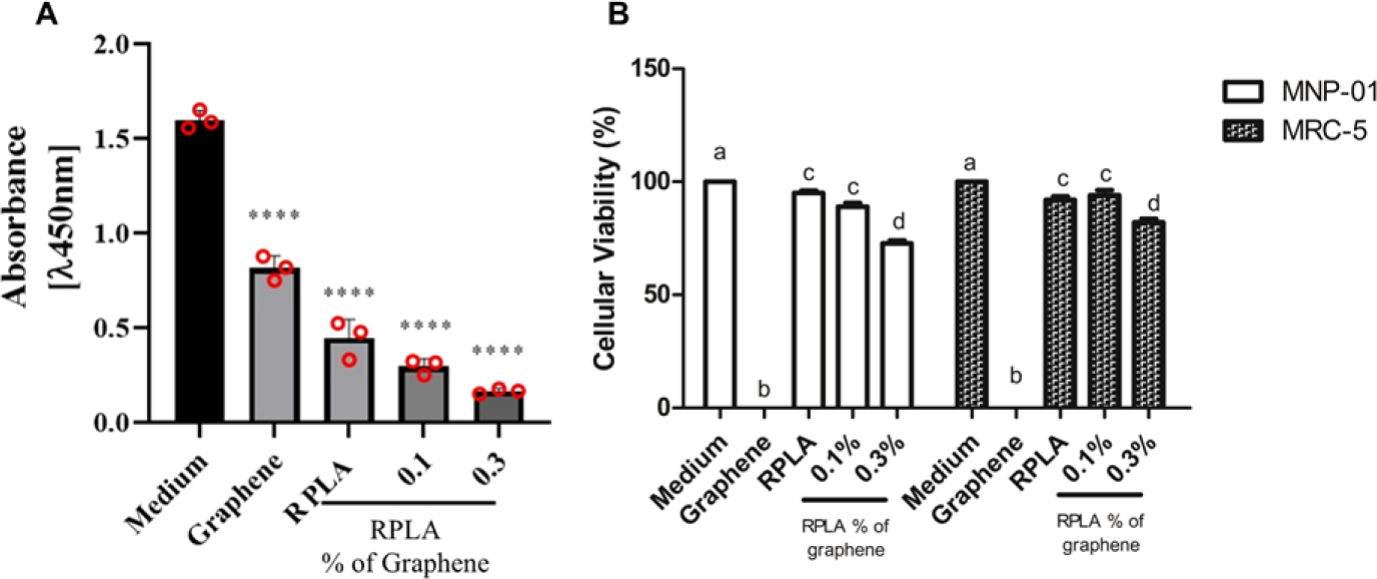
**Cellular Viability of RPLA and RPLAG 0.1–0.3 Samples**. A. Comparative graphs of the viability of melanoma cells (MV3 lineage) on the surface of samples RPLA, RPLA 0.1, and RPLA 0.3. B. cytotoxicity of RPLA, RPLA 0.1, and RPLA 0.3 against two noncancerous cell lines, MNP-01 and MRC-5.

## 4 Discussion

The concentrations of graphene incorporated into the polymer matrix were selected based on reported graphene toxicity thresholds^45^ and on the optical characteristics of the resulting composites. As shown in Figure 2, RPLAG 0.1 samples exhibit partial transparency, while RPLAG 0.3 samples are nearly opaque. This property may be relevant for applications in which visual appearance is critical, such as in dental implants. Regarding the structural profile (Figure 3), all samples display a broad diffraction halo, consistent with the predominantly amorphous nature of PLA-based materials.^46^ The diffractograms of RPLA and RPLAG 0.1 are nearly identical. However, a slight increase in the intensity of the peak around 2θ ≈ 20° and the appearance of a shoulder near 2θ ≈ 26° suggest the contribution of the (002) reflection of multilayer graphene.^47^

The Raman spectra of the samples (Figure 4) exhibit characteristic vibrational bands at 2935 cm^–1^ and 2880 cm^–1^, attributed to the stretching of C–H and CH_3_ bonds, respectively.^48,49^ The band at 1726 cm^–1^ corresponds to the stretching vibration of C=O bonds.^50^ The mode observed near 1460 cm^–1^ is associated with the symmetric bending of CH_3_ groups.^51^ Additional bands at 1350 cm^–1^ and 1300 cm^–1^ are related to the deformation of CH_3_ and C–H groups.^43,48^ Peaks near 1020 cm^–1^ and 1000 cm^–1^ correspond to C–CH_3_ and C–C stretching modes, respectively.^43^ A band around 850 cm^–1^ is attributed to the stretching of ether (C–O–C) bonds.^49^

With respect to surface morphology and elemental distribution, uniformity was observed across all samples regardless of graphene incorporation (Figure 5). In addition to carbon (C), the main element in PLA, the presence of aluminum (Al) and calcium (Ca) was also detected. These elements are likely associated with the composition of the photopolymerizing agent, as they are commonly used alongside photoinitiators to enhance the efficiency of the photopolymerization process.^52,53^

Atomic force microscopy (AFM) analysis revealed a consistent grid-like surface pattern in all samples (Figure 6). This feature is attributed to the stereolithography (SLA) printing process, which employs a Digital Micromirror Device (DMD) composed of thousands of micromirrors. Each micromirror functions as a microaperture, selectively allowing light to pass and polymerize specific regions of the resin.^54,55^ Although the average lateral spacing between valleys in the surface grid remains constant (∼27 μm), the vertical distance between peaks and valleys decreases with the incorporation of graphene into the resin matrix (Figure 6). This parameter may be related to the optical transparency of the resin used in SLA printing, since highly transparent resins exhibit poor absorption in the visible spectrum. As a result, the curing resolution in the Z direction decreases significantly due to impaired light confinement during photopolymerization.^56–58^ The reduction in peak-to-valley amplitude observed in the graphene-containing samples suggests attenuation of the surface grid pattern as the graphene concentration increases. This effect can be attributed to graphene’s role as a nanofiller, primarily due to its high surface area and ability to interfere with light scattering during curing.^59^ This structural change also supports the observed increase in local microhardness, as graphene enhances stress distribution and energy dissipation within the composite.

At the nanometric scale (Figure 7), fluctuations in root-mean-square roughness (Rq) were recorded: 12.32 ± 2.17 nm, 7.66 ± 1.65 nm, and 8.35 ± 0.72 nm for RPLA, RPLAG 0.1, and RPLAG 0.3, respectively. These variations may be linked to factors such as nanoparticle size, interfacial interaction, and the dispersion quality of the filler in the PLA matrix.^60^ An increase in graphene concentration, especially when accompanied by incomplete dispersion, can lead to the formation of nanoclusters due to van der Waals interactions between PLA chains and graphene sheets. These aggregates may act as secondary nanofillers and contribute to alterations in surface roughness parameters.^60–62^

The changes in surface roughness caused by the incorporation of graphene into the PLA resin matrix may also influence the material’s microhardness and modulate cell–surface interactions. Graphene can readily form interfacial interactions due to its high specific surface area.^63,64^ Its electronic structure, characterized by π-electrons oriented perpendicular to the basal plane, enables interactions with electron-rich regions of adjacent molecules, facilitating chemical bonding within the host matrix.^65–67^ The increase in Vickers microhardness (VM) observed for RPLAG 0.3 (Figure 8) may be attributed to improved structural homogeneity, promoted by interfacial bonding between graphene sheets and the resin components. As demonstrated by Heinrich and Vilgis,^68^ increasing the concentration of nanofillers, such as graphene, can lead to a reduction in chain entanglements and cross-linking density, allowing for greater alignment of polymer chains. This configuration promotes a higher local hardness due to increased chain packing, which enhances mechanical resistance and energy dissipation.

The contact angle (Figure 9A), a key parameter for assessing wettability, is directly influenced by both surface roughness and surface energy of the material.^69,70^ In this context, morphological and/or compositional modifications can be associated with variations in wettability. As previously discussed, increasing the graphene content in the polymeric matrix does not result in a linear change in roughness values (Figure 7), suggesting that surface energy plays a more dominant role in modulating the wettability of the PLA/graphene composites.

As observed by Yoon, Ok Ja et al.,^71^ the incorporation of graphene nanosheets into the poly­(D,L-lactic-*co*-glycolic acid) (PLGA) matrix increases the material’s surface energy, thereby enhancing its hydrophilicity. This effect was attributed to a greater contribution from the polar component of surface energy, likely associated with chemical modifications induced by the presence of graphene nanosheets.^71^ Computational studies have shown that graphene exhibits a strong affinity for carboxylic and hydroxyl groups (the terminal groups of PLA chains^72^ due to electrostatic interactions. These interactions lead to the acquisition of a negative surface charge by graphene under both acidic and basic conditions.^59,73,74^ As a result, the material’s polarity increases due to the formation of regions with high charge density, which intensifies with higher graphene content, ultimately enhancing the hydrophilicity of the PLA/graphene nanocomposite.^71,75,76^

From a biophysical perspective, the wettability of a material, along with other factors such as roughness, hydrophilicity, surface energy, and surface charge, plays a critical role in cell adhesion and proliferation. These parameters influence protein adsorption at the cell–material interface, which in turn modulates cellular behavior. Surface functional groups introduced by the incorporation of graphene are especially important in defining these characteristics, as they can alter the physicochemical properties of the interface and affect cell–substrate interactions.^77,78^

Arima, Yusuke, and Iwata^79^ reported that healthy cells exhibit optimal adhesion on surfaces with intermediate levels of hydrophobicity and hydrophilicity. Similarly, Feng, Jing, and Zhiguang Guo^80^ emphasized graphene’s ability to function as a surface coating that modulates a material’s wettability. These findings reinforce the notion that graphene possesses a high capacity to modify the interfacial properties of polymers, extending its influence beyond mechanical and electrical enhancements to include physicochemical surface behavior.

Since the characteristics of membrane proteins can vary depending on the cell type,^81^ the intrinsic nature of the cell is also a determining factor in its affinity for material surfaces.^82^ In this study, it was observed that MV3 melanoma cells exhibited low affinity for surfaces with high wettability (i.e., high surface energy), and this affinity decreased further with the incorporation of multilayer graphene into the PLA matrix, as shown in Figure 10.

When considering materials for medical applications, their interaction with a key biological structure (cells) is of central importance. This interaction is mediated by the plasma membrane, which defines the cell boundary and plays a critical role in processes such as substance transport, intercellular communication, and cell adhesion.^83^ Understanding how materials interface with the plasma membrane is essential for designing biomaterials capable of eliciting desired biological responses.

A key feature of cell membranes is their surface charge, which plays a crucial role in mediating cellular interactions, including adhesion, intercellular signaling, and cell–material interfaces.^83^ The sodium–potassium pump (Na^+^/K^+^ -ATPase) actively transports ions across the membrane, maintaining distinct intracellular and extracellular concentrations of Na^+^ and K^+^. This active transport generates an electrochemical gradient, establishing a transmembrane potential and contributing to the accumulation of surface charges. Additionally, ion channels selectively regulate ion flow in response to electrochemical gradients. Their opening and closing modulate charge distribution across the membrane and are essential in maintaining and altering the membrane potential.

In our in vitro assays, we observed a decrease in the viability of tumor cells as the graphene content in the polymer matrix increased (Figure 10A). In contrast, nontumor cells maintained high viability, suggesting a selective biological response of the PLA/graphene composites. Cell–surface interactions are inherently complex and influenced by multiple material characteristics, including chemical composition, biocompatibility, surface charge, roughness, and wettability (or surface free energy).^84^ In this context, the reduced affinity of MV3 melanoma cells for PLA surfaces containing graphene may be attributed to modifications in these properties induced by graphene incorporation, as previously discussed.

Cancer cells typically exhibit a negative surface charge.^85,86^ Similarly, graphene and its derivatives tend to acquire negative surface charge when dispersed in polymer matrices due to interactions with electronegative organic functional groups.^87,88^ Consequently, electrostatic repulsion between the negatively charged cell membranes and material surfaces may hinder protein adsorption and subsequent adhesion. By contrast, nontumor cells (MNP-01 and MRC-5), which may possess a different surface charge distribution, appear to exhibit greater affinity for the material, likely due to reduced electrostatic repulsion.

In addition to altering surface charge, graphene also modifies the surface roughness of the material, as shown in Figures 6 and 7. Surface roughness is a critical factor influencing cell adhesion, proliferation, and differentiation.^89^ As reported by Majhy et al.,^84^ nontumor cells show reduced growth on surfaces with either very low (<20 nm) or moderately high (20–40 nm) roughness, as membrane elongation becomes restricted, thereby impairing the cells’ ability to attach effectively.

Another important parameter affecting cellular behavior is substrate elasticity. Cells are capable of sensing the mechanical properties of the surface by applying forces derived from cytoskeletal reorganization during structural adaptation.^90^ However, the cellular response to surface stiffness may vary depending on the cell type. In general, substrates with moderate stiffness tend to promote better cell proliferation and differentiation.^91^ For instance, poly­(dimethylsiloxane) (PDMS), a commonly used substrate for supporting cell growth and culture,^92^ exhibits a typical elastic modulus of approximately 1000 kPa.^84^

Therefore, considering that the roughness and hardness parameters of the RPLAG 0.1 and RPLAG 0.3 samples are relatively similar, the observed reduction in MV3 cell viability and proliferation is likely related to decreased cell adhesion. This effect may be attributed to changes in the polymer’s net surface charge, which is induced by graphene incorporation.

## 5 Conclusions

This study demonstrates that resin-based 3D printing, particularly via stereolithography (SLA), is a promising technique due to its high resolution, processing simplicity, and ease of resin functionalization. The fabrication of PLA/graphene composites exemplifies how material properties can be tailored to meet the specific demands of biomedical applications.

The characterization results revealed that incorporating graphene at concentrations of 0.1 and 0.3 wt % significantly alters the physical properties of the printed composites. These changes include increased microhardness, modulation of surface roughness, variations in the amorphous halo intensity, and distinctive vibrational signatures associated with organic groups. Graphene’s presence appears to enhance the resin’s curing efficiency and surface quality, contributing to a more uniform ultrastructure. These characteristics are closely associated with the observed selective inhibition of MV3 tumor cell proliferation in vitro, likely driven by surface charge distribution and topographical modifications induced by graphene.

Taken together, these findings highlight the potential of PLA/graphene nanocomposites for biomedical applications, particularly in systems aimed at modulating tumor cell behavior. Moreover, SLA 3D printing has proven to be a reliable and versatile processing method, combining cost-effectiveness with excellent resolution and compositional control.

## Supplementary Material



## Notes

Detailed synthesis methodology and characterization of graphene used for resin functionalization. The step-by-step electrochemical exfoliation protocol, as well as X-ray diffraction (XRD) and Raman spectroscopy data confirming the successful production of multilayer graphene with low structural defect density (PDF)
