# Supplementary material for: Sustainable Graphene Synthesis and Analysis of Graphene-Based PLA Nanocomposites: Impacts of Polymer Functionalization and Potential Applications in Cancer Treatments
Source: ACS Omega. 2025 Jun 5;10(23):24520–31. doi: 10.1021/acsomega.5c01094 (PMC12177593; doi:10.1021/acsomega.5c01094)
Supplement: Supplementary file 1 [file ao5c01094_si_001.pdf]

SUPPLEMENTARY MATERIAL

Sustainable Graphene Synthesis and Analysis of Graphene-Based PLA Nanocomposites: Impacts of Polymer Functionalization and Potential Applications in Cancer Treatments

Álefe Roger Silva França[a,b], Beatriz da Silva Batista[a,b], Joel Félix Silva Diniz Filho[b,c], Rosa Maria Viana Sousa[b], Alan Silva de Menezes[a,b], Clenilton Costa dos Santos[b], Ralph Santos-Oliveira[d,e], Pedro Filho Noronha Souza[f], Luzeli Moreira da Silva[a], Luciana Magalhães Rebêlo Alencar[a,b*]

[a] Center for Social Sciences, Health and Technology, Federal University of Maranhão, Advanced Unit, Imperatriz, MA, Brazil; [b] Department of Physics, Laboratory of Biophysics and Nanosystems, Federal University of Maranhão, Campus Bacanga, São Luís, MA, Brazil; [c] Coordination of the Bachelor's Degree In Natural Sciences – Physics/CCBA, Federal University of Maranhão, Campus Bacabal, Bacabal, MA, Brazil; [d] Brazilian Nuclear Energy Commission, Nuclear Engineering Institute, Laboratory of Nanoradiopharmacy and Synthesis of Novel Radiopharmaceuticals, Rio de Janeiro, Rio de Janeiro, Brazil;[e] Laboratory of Radiopharmacy and Nanoradiopharmaceuticals, Rio de Janeiro State University, Rio de Janeiro, Brazil; [f] Visiting Researcher at the Cearense Foundation to Support Scientific and Technological Development, Fortaleza 60325-452, CE, Brazil.

***Corresponding author:** e-mail: luciana.alencar@ufma.br; telephone: +55 98 8407-0117; postal address: Av. dos Portugueses, 1966 - Vila Bacanga, São Luís - MA, 65080-805, Universidade Federal do Maranhão, Laboratório de Biofísica e Nanosistemas.*

OVERVIEW

The supplementary material provides additional details not included in the main manuscript due to space limitations. It includes the following sections:

1. Motivation;
2. Detailed methodology for graphene synthesis (see Section 2);
3. Additional results from graphene characterization (see Section 3);
4. Final considerations.

1. Motivation

Graphene has attracted significant attention due to its exceptional properties and its potential in the development of polymer matrix composites. It is a two-dimensional crystalline material composed of carbon atoms arranged in a hexagonal lattice. Graphene is considered an allotropic form of carbon, derived directly from graphite (its bulk counterpart), which consists of multiple graphene layers stacked via weak van der Waals forces. In graphite, each carbon atom is sp^2-hybridized, forming a continuous network of covalent bonds within each layer [1].

Each carbon atom in the basal plane of graphene is bonded to its neighbors through strong covalent σ-bonds, which provide the material with high mechanical strength and structural stability [2]. In contrast, the stacking of graphene layers in graphite is maintained by weak van der Waals interactions. These weak interlayer forces result in low shear strength, facilitating the exfoliation of individual graphene sheets.

Graphene's high electrical and thermal conductivity is primarily attributed to the delocalized electrons in π molecular orbitals, which arise from non-hybridized orbitals oriented perpendicular to the basal plane. As previously noted, these π orbitals have relatively low binding energy, facilitating electron mobility and favoring conduction mechanisms.

The increased ease of electron delocalization in graphene is largely attributed to its two-dimensional confinement [3], which results in electronic behavior conducive to exceptional electrical, thermal, and optical properties. This has earned graphene its reputation as a "material of the future."

Owing to this two-dimensional confinement and its hexagonal crystal structure, electrons in graphene behave as though they possess an extremely low effective mass, resembling massless Dirac fermions. This phenomenon leads to remarkably high electronic mobility, due to the weak interaction between the charge carriers and the positively charged atomic lattice [4].

With the advancement of synthesis techniques that allow graphene and its derivatives to be produced on a larger scale, these materials have increasingly been incorporated into various systems, including polymer matrices. Graphene has been shown to enhance polymers' mechanical, thermal, and electrical properties [5,6], further broadening their application potential, particularly in the biomedical field.

Given that polymers are typically electrical insulators, incorporating graphene into their structure offers a more sustainable and cost-effective strategy for producing conductive materials, particularly in applications such as printed electronic circuits [7]. The ability of graphene to alter the electrical behavior of polymers stems from its high interfacial reactivity and capacity to form strong interactions with surrounding matrices. Additionally, its influence on the modulation of net surface charge is a key factor in the context of biological applications.

From a biological perspective, graphene and its derivatives have been extensively explored for a wide range of applications, as illustrated in Figure S1. Owing to the presence of oxygen-containing functional groups, graphene oxide (GO) can be readily functionalized with various molecules, such as polyethylene glycol (PEG) [8] and Pluronic F-127 [9], resulting in versatile nanomaterials suitable for drug delivery and biomedical packaging applications [10].

Graphene has also been investigated in tissue engineering to create smart interfaces that promote cell adhesion, proliferation, and differentiation [11,12]. Among its derivatives, graphene quantum dots (GQDs) stand out due to their low toxicity, excellent solubility, and stable photoluminescence, making them promising candidates for biosensing and bioimaging applications [13].

Another important property of graphene is its antibacterial activity, which is attributed to multiple mechanisms, including physical damage to bacterial membranes by sharp edges, oxidative stress induced by charge transfer, and the generation of reactive oxygen species (ROS) [14,15].

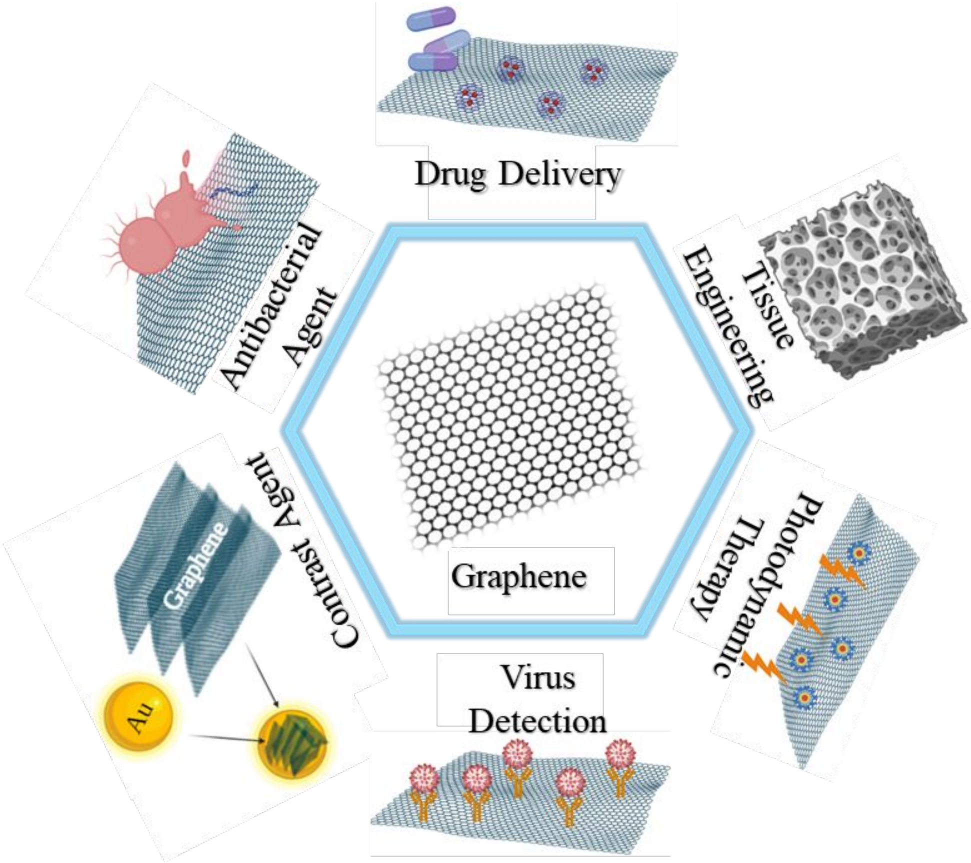

Figure S1. Applications of graphene and its derivatives in biological systems. Graphene-based materials have been employed in a wide range of biomedical applications due to their biocompatibility and tunable surface properties.

2. Graphene synthesis

Different synthesis routes were tested, primarily varying in the type of graphite used as the precursor. The main method adopted and optimized in this study was electrochemical exfoliation. This technique involves using an electrolytic solution to mediate ionic intercalation between graphite layers, weakening interlayer van der Waals forces and promoting exfoliation. As a result, graphene sheets are produced through the disruption of interplanar interactions.

The synthesis procedure began with the selection of an appropriate solvent for the electrolytic solution. Several electrochemical exfoliation methods described in the literature involve the use of strong inorganic acids to facilitate ionic intercalation. However, such routes (particularly those employing acids like sulfuric acid (H_2SO_4) [16]) are not ideal for biomedical applications, as residual acid traces may remain in the final product and pose cytotoxic risks to cells and tissues. To address this concern, the methodology developed in this study employed sodium sulfate (Na_2SO_4), a neutral inorganic salt, as the electrolyte solute [16].

Following the selection of the electrolytic solution, the next step was to determine the appropriate materials for the cathode and anode in the electrochemical cell. Initial tests using graphite sheets for both electrodes were conducted; however, alternative materials such as platinum (Pt) and nickel (Ni) were later selected to improve the purity and structural quality of the synthesized graphene. At this stage, pencil graphite was also chosen as the source material. Its composite nature—graphite mixed with clay—proved advantageous for the exfoliation process. During exfoliation, non-exfoliated graphite and clay residues tend to settle at the bottom of the reaction vessel, aiding in the separation of exfoliated graphene [17]. Additionally, the presence of clay serves as a modulating agent, helping to regulate the exfoliation rate and prevent abrupt reactions that could lead to the formation of large aggregates or multilayer graphite flakes.

The experimental procedure began with the preparation of the electrolytic cell in a 250 mL beaker. A solution was prepared by dissolving 10.65 g of sodium sulfate (Na_2SO_4) in 75 mL of distilled water, followed by homogenization using a magnetic stirrer. The electrodes were then fixed to the inner walls of the beaker using adhesive tape. A graphite rod was connected to the positive terminal of a power supply (anode), and a platinum wire was connected to the negative terminal (cathode). A potential difference of 10 V was applied for 15 minutes, or until complete exfoliation of the graphite was observed (Figure S2).

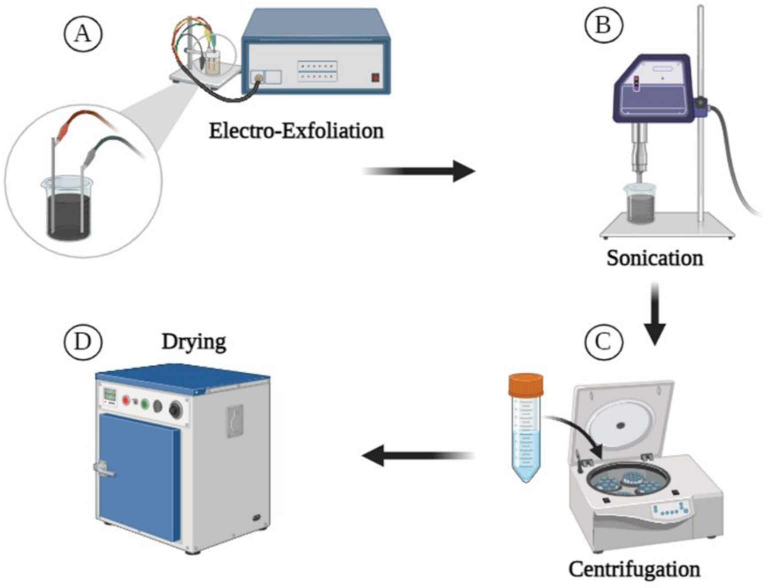

Figure S2. Experimental procedure for graphene synthesis. A. Electrochemical exfoliation of graphite in a sodium sulfate solution. B. Ultrasonication using a tip sonicator to promote mechanical exfoliation and further separation of layers. C. Sample washing step, involving three rinses with distilled water. D. Drying of the resulting graphene in a laboratory oven.

Following exfoliation, the dispersion underwent ultrasonication to promote further mechanical separation of the graphene layers (Figure S2). A tip sonicator operating at 40 kHz was applied for 30 minutes. After sonication, the material was subjected to a purification step involving centrifugation at 4000 rpm for 30 minutes. This process concentrated the exfoliated graphene at the bottom of the Falcon tube, allowing the supernatant to be removed. Distilled water was added to the remaining material, and the centrifugation cycle was repeated four times to ensure thorough washing.

The final stage of the synthesis involved drying the exfoliated material. All graphene obtained was transferred to a Petri dish and dried in a laboratory oven at 90 °C for 30 minutes. After drying, the samples were subjected to structural characterization by X-ray diffraction (XRD) and Raman spectroscopy (RS).

The entire synthesis process was tested under various experimental conditions, particularly by modifying the configuration of the electrolytic cell. Graphite rods from pencils with varying clay content were used, specifically grades 8B, 6B, 2B, and HB. The general clay content ranking follows the order: 8B < 6B < 2B < HB. Although the graphite-to-clay ratio may vary by manufacturer, typical values are: 8B (90–95% graphite), 6B (80–90%), 2B (70–80%), and HB (60–70%) [18].

Additionally, in some experiments, the platinum electrode was replaced with nickel. These tests were critical for optimizing the system and selecting the conditions that yielded graphene with high purity and low structural defect density.

3. Graphene characterization

The graphene samples obtained through different synthesis routes were characterized by X-ray diffraction (XRD). Figure S3 shows the diffractograms of these samples, highlighting variations primarily related to the type of graphite precursor used during electrochemical exfoliation.

XRD analysis was performed to assess the effectiveness of the exfoliation process. The characteristic peaks at approximately 2θ ≈ 26.6° and 54.4° correspond to the (002) and (004) crystallographic planes of graphite, respectively [19–21]. The graphite types employed as anodes in the electrolytic cell varied in clay content, as they originated from pencil graphite (a graphite–clay composite). Due to its quasi-two-dimensional structure, multilayer graphene exhibits a distinct diffraction pattern compared to bulk graphite, as illustrated in Figure S3.

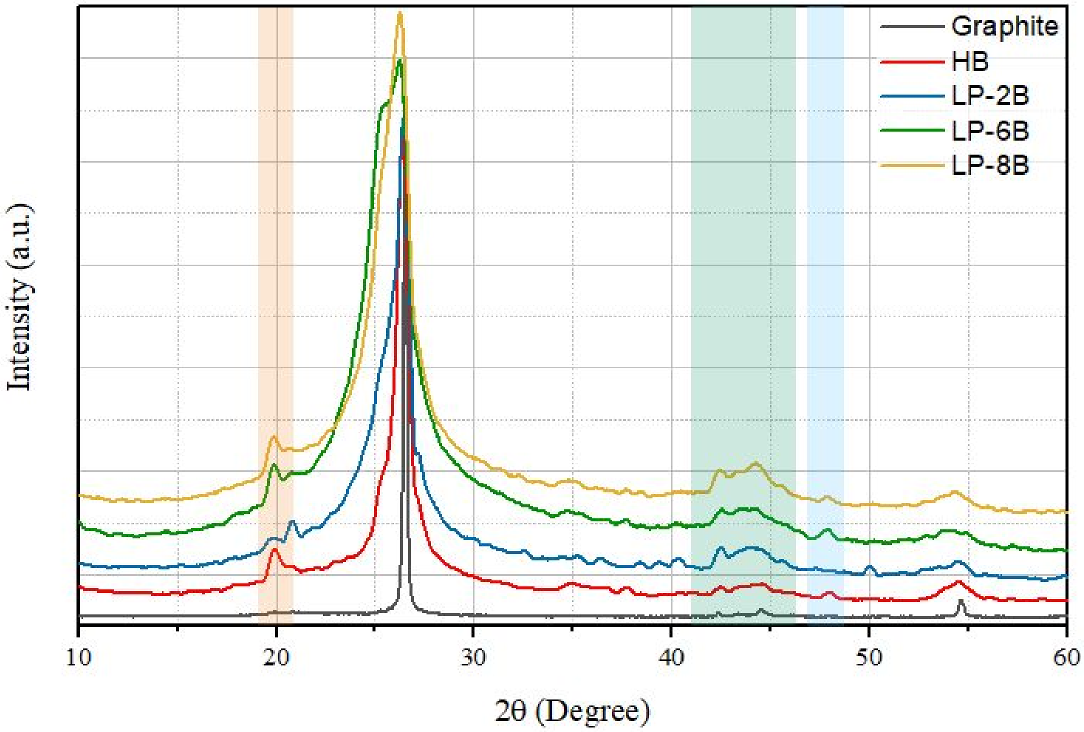

Figure S3. Multilayer Graphene diffractogram. Comparison of the diffractograms of graphene samples synthesized from the electrochemical exfoliation method using different precursors.

The orange-shaded region in Figure S3 highlights a peak near 2θ ≈ 19.8°, which is associated with clay species, particularly of the montmorillonite type [17,22]. After exfoliation, graphene tends to concentrate at the top of the suspension, while heavier components, such as graphite flakes and nanoclay aggregates, settle at the bottom. The presence of a weak clay-related peak in the diffractogram indicates that only a minimal amount of nanoclay remained associated with the exfoliated graphene. However, this residual content did not induce significant structural changes, as confirmed by the Raman spectrum of the graphene sample obtained using the LP-8B route (Figure S4).

The higher intensity of graphite peaks compared to those of graphene can be attributed to its highly ordered crystalline structure [20,21]. In contrast, the presence of defects in the basal planes and edges of graphene layers reduces the material's overall crystallinity, which is also reflected in the broadening of the diffraction peaks. As the clay content in the graphite precursor decreases, the resulting samples exhibit peak broadening at $2\theta \approx 26.6°$ and $2\theta \approx 54.4°$, along with a slight shift to lower angles. These features may be associated with the removal of structural impurities and more effective exfoliation of graphite layers, leading to the formation of nanometric graphene (~20 nm) [23].

An additional indication supporting the purity of the synthesized material is the absence of a characteristic peak around $2\theta \approx 11.6°$, typically associated with oxygen-containing functional groups in graphene oxide [24]. This confirms that the material obtained is not graphene oxide.

Based on the synthesis and processing conditions, multilayer graphene can exhibit different stacking orders, primarily the 2H (hexagonal, ABA) and 3R (rhombohedral, ABC) phases, which are considered polymorphic forms of multilayer graphene [24,25]. The 2H phase is the most commonly observed in graphene produced via mechanical exfoliation or chemical vapor deposition (CVD). In contrast, the 3R phase is less frequent and typically arises under more controlled synthesis conditions, such as fine-tuned CVD parameters or specific thermal treatments [26]. These stacking configurations significantly influence the structural and electronic properties of multilayer graphene. As reported by Low, It-Meng, et al. [21], the presence of the 3R stacking structure imparts semiconducting behavior to graphene, enabling the modulation of its bandgap.

In this context, the diffractograms of the samples obtained via the HB, LP-2B, LP-6B, and LP-8B routes exhibit common peaks in the region between $2\theta \approx 40°$ and 62°, particularly within the shaded areas in Figure S3. These peaks can be attributed to reflections from both the 2H and 3R phases. For the HB route, more intense peaks are observed at $2\theta \approx 54.94°$ and 62.26°, corresponding to the 2H(004) and 3R(10-5) planes, respectively. The LP-2B sample displays peaks at $2\theta \approx 42.35°$ (2H(100)), 44.30° (2H(101)), 50.02° (2H(102)), and 54.59° (2H(004)) [21,26]. Similarly, the LP-8B sample exhibits peaks at $2\theta \approx 42.46°$, 44.23°, and 54.39°, which are assigned to the 2H(100), 2H(101), and 2H(004)/3R(006) planes, respectively [27].

Figure S4 shows the Raman spectrum of the graphene sample synthesized using the methodology developed in this study, in which 8B graphite was employed as the precursor material for electrochemical exfoliation. The spectrum exhibits characteristic peaks corresponding to the G band (~1580 cm^{-1}), D band (~1350 cm^{-1}) [28], and D′ band (~1620 cm^{-1}), all of which are indicative of graphitic carbon structures. A broadband centered at ~2716 cm^{-1} is also observed, corresponding to the 2D band [23], a key feature for identifying few-layer graphene. The G and D bands are signatures of carbon-based materials and are found in all polyaromatic hydrocarbons [29]. These bands are particularly important in graphene, as they provide information about structural order and the presence of defects [30]. Additional bands are also present in the spectrum, including the G* band at ~2450 cm^{-1} and the D″ band at ~3242 cm^{-1}.

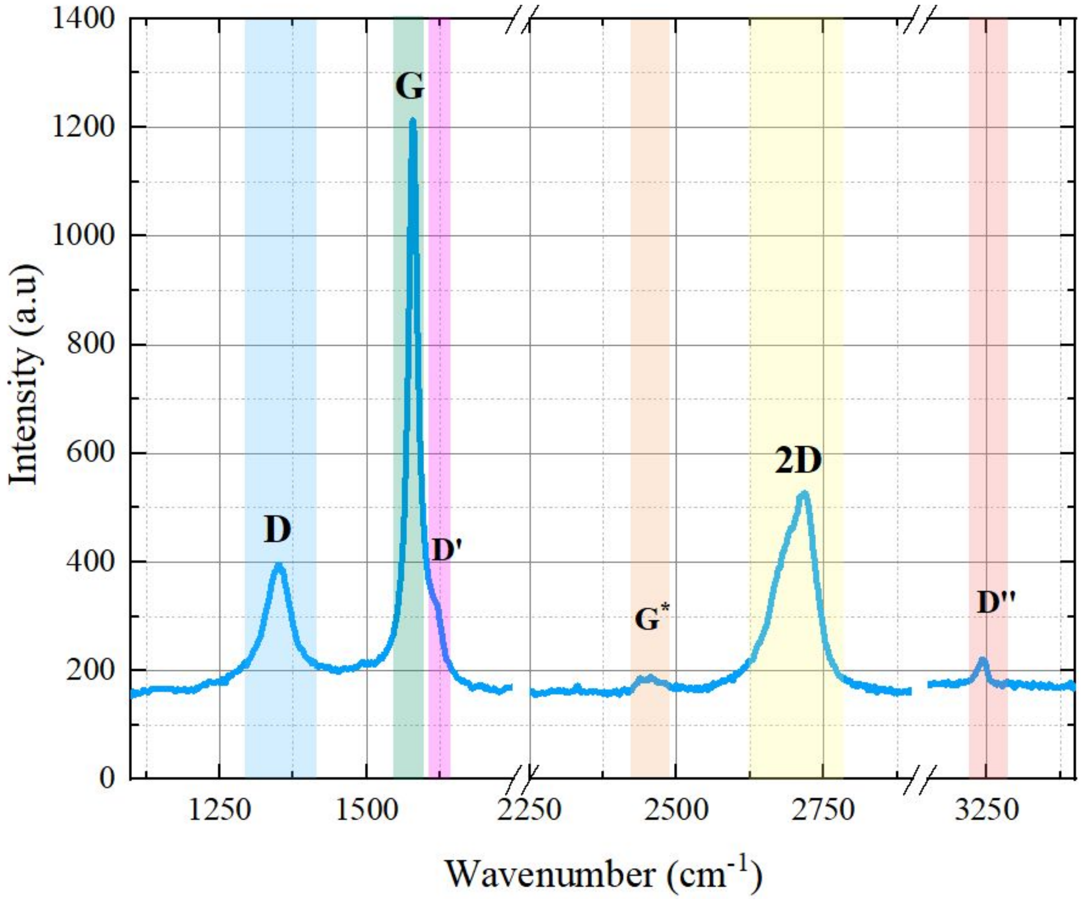

Figure S4. Raman spectrum of multilayer graphene. The spectrum corresponds to the graphene sample obtained using the developed synthesis methodology, in which 8B pencil graphite was employed as the anode in the electrochemical cell.

The G band is considered the primary Raman feature of graphene, as it is the only band associated with first-order phonon scattering [30]. It corresponds to the in-plane stretching vibrations of C–C bonds in sp^2-hybridized carbon atoms arranged in a hexagonal lattice [31]. A prominent G band is typically associated with high structural quality in graphitic materials [32]. In contrast, the D band arises from breathing-like vibrational modes of sp^2 carbon atoms within six-membered aromatic rings and is activated by the presence of structural defects [29,33,34]. Therefore, the intensity of the D band serves as an indicator of the defect density in the graphene lattice. Defects also give rise to the D′ band, which—unlike the D band—does not require a double-resonance mechanism for activation [30].

The 2D band arises from a second-order double-resonance Raman process and is closely linked to the electronic structure of graphene. Its position and intensity are commonly used to distinguish monolayer graphene from multilayer forms [30,35,36]. As reported by Park et al. [36], in multilayer graphene, the 2D band shifts to higher wavenumbers compared to monolayer graphene and exhibits reduced intensity as the number of layers increases. Furthermore, Lui et al. [37] demonstrated that the shape of the 2D band can also be used to identify different stacking configurations in multilayer graphene.

Similar to the 2D band, the G* band also arises from a double-resonance Raman scattering process. However, it involves a combination of an in-plane transverse optical (iTO) phonon and a longitudinal acoustic (LA) phonon [38,39]. As reported in reference [40], the intensity of the G* band tends to decrease with increasing graphene layer number. The D″ band, in turn, is attributed to a second-order resonance process involving the D′ mode [31].

Since the intensities of the G and D bands vary inversely with the crystallite size and the distance between defects [41], the intensity ratio I_D/I_G is a key parameter for evaluating the structural quality of graphene. This ratio provides insight into the degree of disorder in the material, including the presence of edges and oxygen-containing functional groups [3,4,42]. From the Raman spectrum, the intensities of the D and G bands were measured as 377.46 and 2172.41, respectively, resulting in an I_D/I_G ratio of 0.17. According to Das et al. [43], lower I_D/I_G values (approaching zero) indicate fewer structural defects and higher crystalline quality. Therefore, the obtained result ($I_D/I_G < 0.45$) suggests that the synthesized graphene has a low defect density and retains key structural features associated with high-quality multilayer graphene [16].

4. Final considerations

X-ray diffraction (XRD) played a crucial role in validating the developed graphene synthesis route, which successfully yielded nanometric graphene. These results enabled refinement of the methodology and helped identify the optimal conditions for producing high-purity material.

Raman spectroscopy was also fundamental in assessing the structural quality of the graphene obtained. The presence of the characteristic G and D bands confirmed the graphitic nature of the material. Moreover, the low I_D/I_G ratio indicated a low density of structural defects. Combined with the XRD results, these findings suggest that the synthesized graphene contains minimal oxygen-based functional groups.

Raman analysis further contributed to understanding the composition of the PLA-based resin samples. The vibrational signatures of the organic groups became more pronounced with increasing graphene content, and additional bands emerged, confirming the successful incorporation of graphene into the polymer matrix.

References

[1] J.H. Warner, F. Schaffel, M. Rummeli, A. Bachmatiuk, Graphene: Fundamentals and Emergent Applications, Newnes, 2012.

[2] H. Aoki, M.S. Dresselhaus, Physics of Graphene, Springer Science & Business Media, 2013.

[3] A.K. Geim, K.S. Novoselov, The rise of graphene, Nature Mater 6 (2007) 183–191. https://doi.org/10.1038/nmat1849.

[4] K.S. Novoselov, A.K. Geim, S.V. Morozov, D. Jiang, M.I. Katsnelson, I.V. Grigorieva, S.V. Dubonos, A.A. Firsov, Two-dimensional gas of massless Dirac fermions in graphene, Nature 438 (2005) 197–200.

[5] R.S. Lodhi, P. Kumar, A. Achuthanunni, M. Rahaman, P. Das, 3 - Mechanical properties of polymer/graphene composites, in: M. Rahaman, L. Nayak, I.A. Hussein, N.C. Das (Eds.), Polymer Nanocomposites Containing Graphene, Woodhead Publishing, 2022: pp. 75–105. https://doi.org/10.1016/B978-0-12-821639-2.00019-7.

[6] P.N. Khanam, D. Ponnamma, M.A. AL-Madeed, Electrical Properties of Graphene Polymer Nanocomposites, in: K.K. Sadasivuni, D. Ponnamma, J. Kim, S. Thomas (Eds.), Graphene-Based Polymer Nanocomposites in Electronics, Springer International Publishing, Cham, 2015: pp. 25–47. https://doi.org/10.1007/978-3-319-13875-6_2.

[7] D. Zhang, B. Chi, B. Li, Z. Gao, Y. Du, J. Guo, J. Wei, Fabrication of highly conductive graphene flexible circuits by 3D printing, Synthetic Metals 217 (2016) 79–86. https://doi.org/10.1016/j.synthmet.2016.03.014.

[8] Z. Liu, J.T. Robinson, X. Sun, H. Dai, PEGylated nanographene oxide for delivery of water-insoluble cancer drugs, J Am Chem Soc 130 (2008) 10876–10877. https://doi.org/10.1021/ja803688x.

[9] H. Hu, J. Yu, Y. Li, J. Zhao, H. Dong, Engineering of a novel pluronic F127/graphene nanohybrid for pH responsive drug delivery, Journal of Biomedical Materials Research Part A 100 (2012) 141–148.

[10] K. Yang, L. Feng, Z. Liu, The advancing uses of nano-graphene in drug delivery, Expert Opinion on Drug Delivery 12 (2015) 601–612. https://doi.org/10.1517/17425247.2015.978760.
[11] M. Bramini, G. Alberini, E. Colombo, M. Chiacchiaretta, M.L. DiFrancesco, J.F. Maya-Vetencourt, L. Maragliano, F. Benfenati, F. Cesca, Interfacing Graphene-Based Materials With Neural Cells, Front. Syst. Neurosci. 12 (2018). https://doi.org/10.3389/fnsys.2018.00012.
[12] C. Nie, L. Ma, S. Li, X. Fan, Y. Yang, C. Cheng, W. Zhao, C. Zhao, Recent progresses in graphene based bio-functional nanostructures for advanced biological and cellular interfaces, Nano Today 26 (2019) 57–97. https://doi.org/10.1016/j.nantod.2019.03.003.
[13] S. Chung, R.A. Revia, M. Zhang, Graphene quantum dots and their applications in bioimaging, biosensing, and therapy, Advanced Materials 33 (2021) 1904362.
[14] W. Hu, C. Peng, W. Luo, M. Lv, X. Li, D. Li, Q. Huang, C. Fan, Graphene-based antibacterial paper, ACS Nano 4 (2010) 4317–4323.
[15] P. Kumar, P. Huo, R. Zhang, B. Liu, Antibacterial properties of graphene-based nanomaterials, Nanomaterials 9 (2019) 737.
[16] F. Liu, C. Wang, X. Sui, M.A. Riaz, M. Xu, L. Wei, Y. Chen, Synthesis of graphene materials by electrochemical exfoliation: Recent progress and future potential, Carbon Energy 1 (2019) 173–199. https://doi.org/10.1002/cey2.14.
[17] K. Chen, D. Xue, S. Komarneni, Nanoclay assisted electrochemical exfoliation of pencil core to high conductive graphene thin-film electrode, Journal of Colloid and Interface Science 487 (2017) 156–161. https://doi.org/10.1016/j.jcis.2016.10.028.
[18] M.C. Sousa, J.W. Buchanan, Observational Models of Graphite Pencil Materials, Computer Graphics Forum 19 (2000) 27–49. https://doi.org/10.1111/1467-8659.00386.
[19] M.J. McAllister, J.-L. Li, D.H. Adamson, H.C. Schniepp, A.A. Abdala, J. Liu, M. Herrera-Alonso, D.L. Milius, R. Car, R.K. Prud'homme, I.A. Aksay, Single Sheet Functionalized Graphene by Oxidation and Thermal Expansion of Graphite, Chem. Mater. 19 (2007) 4396–4404. https://doi.org/10.1021/cm0630800.
[20] B. Andonovic, A. Grozdanov, P. Paunović, A.T. Dimitrov, X-ray diffraction analysis on layers in graphene samples obtained by electrolysis in molten salts: a new perspective, Micro & Nano Letters 10 (2015) 683–685. https://doi.org/10.1049/mnl.2015.0325.
[21] I.-M. Low, H.M. Albetran, M. Degiorgio, Structural Characterization of Commercial Graphite and Graphene Materials, J Nanotechnol Nanomaterials Volume 1 (2020) 23–30. https://doi.org/10.33696/Nanotechnol.1.005.
[22] Z. Wu, H. Zhao, X. Zhou, Y. Wang, K. Zuo, H. Cheng, Thermal Migration Behavior of Na+, Cu2+ and Li+ in Montmorillonite, Minerals 12 (2022) 477.
[23] R. Hack, C.H.G. Correia, R.A.D.S. Zanon, S.H. Pezzin, Characterization of graphene nanosheets obtained by a modified Hummer's method, Matéria (Rio J.) 23 (2018). https://doi.org/10.1590/s1517-707620170001.0324.
[24] R. Siburian, H. Sihotang, S.L. Raja, M. Supeno, C. Simanjuntak, New route to synthesize of graphene nano sheets, Oriental Journal of Chemistry 34 (2018) 182.
[25] H. Bergeron, D. Lebedev, M.C. Hersam, Polymorphism in post-dichalcogenide two-dimensional materials, Chemical Reviews 121 (2021) 2713–2775.
[26] F. Pan, K. Ni, Y. Ma, H. Wu, X. Tang, J. Xiong, Y. Yang, C. Ye, H. Yuan, M.-L. Lin, Phase-changing in graphite assisted by interface charge injection, Nano Letters 21 (2021) 5648–5654.
[27] M.S. Seehra, U.K. Geddam, D. Schwegler-Berry, A.B. Stefaniak, Detection and quantification of 2H and 3R phases in commercial graphene-based materials, Carbon 95 (2015) 818–823. https://doi.org/10.1016/j.carbon.2015.08.109.

[28] K. Parvez, R. Li, S.R. Puniredd, Y. Hernandez, F. Hinkel, S. Wang, X. Feng, K. Müllen, Electrochemically Exfoliated Graphene as Solution-Processable, Highly Conductive Electrodes for Organic Electronics, ACS Nano 7 (2013) 3598–3606. https://doi.org/10.1021/nn400576v.
[29] C. Castiglioni, F. Negri, M. Rigolio, G. Zerbi, Raman activation in disordered graphites of the A1′ symmetry forbidden k≠0 phonon: The origin of the D line, The Journal of Chemical Physics 115 (2001) 3769–3778. https://doi.org/10.1063/1.1381529.
[30] L.M. Malard, M.A. Pimenta, G. Dresselhaus, M.S. Dresselhaus, Raman spectroscopy in graphene, Physics Reports 473 (2009) 51–87. https://doi.org/10.1016/j.physrep.2009.02.003.
[31] A.C. Ferrari, Raman spectroscopy of graphene and graphite: Disorder, electron–phonon coupling, doping and nonadiabatic effects, Solid State Communications 143 (2007) 47–57. https://doi.org/10.1016/j.ssc.2007.03.052.
[32] A. Jorio, E.H.M. Ferreira, M.V. Moutinho, F. Stavale, C.A. Achete, R.B. Capaz, Measuring disorder in graphene with the G and D bands, Physica Status Solidi (b) 247 (2010) 2980–2982.
[33] I.A. Popov, K.V. Bozhenko, A.I. Boldyrev, Is graphene aromatic?, Nano Res. 5 (2012) 117–123. https://doi.org/10.1007/s12274-011-0192-z.
[34] A.C. Ferrari, J. Robertson, Interpretation of Raman spectra of disordered and amorphous carbon, Physical Review B 61 (2000) 14095.
[35] C. Thomsen, S. Reich, Double resonant Raman scattering in graphite, Physical Review Letters 85 (2000) 5214.
[36] J. Park, A. Reina, R. Saito, J. Kong, G. Dresselhaus, M. Dresselhaus, G′ band Raman spectra of single, double and triple layer graphene, Carbon 47 (2009) 1303–1310.
[37] C.H. Lui, Z. Li, Z. Chen, P.V. Klimov, L.E. Brus, T.F. Heinz, Imaging stacking order in few-layer graphene, Nano Letters 11 (2011) 164–169.
[38] D. Mafra, G. Samsonidze, L. Malard, D.C. Elias, J. Brant, F. Plentz, E.S. Alves, M.A. Pimenta, Determination of LA and TO phonon dispersion relations of graphene near the Dirac point by double resonance Raman scattering, Physical Review B—Condensed Matter and Materials Physics 76 (2007) 233407.
[39] J. Maultzsch, S. Reich, C. Thomsen, Double-resonant Raman scattering in graphite: Interference effects, selection rules, and phonon dispersion, Physical Review B—Condensed Matter and Materials Physics 70 (2004) 155403.
[40] D. Yoon, H. Cheong, Raman spectroscopy for characterization of graphene, Raman Spectroscopy for Nanomaterials Characterization (2012) 191–214.
[41] C. Casiraghi, A. Hartschuh, H. Qian, S. Piscanec, C. Georgi, A. Fasoli, K.S. Novoselov, D.M. Basko, A.C. Ferrari, Raman Spectroscopy of Graphene Edges, Nano Lett. 9 (2009) 1433–1441. https://doi.org/10.1021/nl8032697.
[42] F. Liu, S. Song, D. Xue, H. Zhang, Folded structured graphene paper for high performance electrode materials, Adv Mater 24 (2012) 1089–1094. https://doi.org/10.1002/adma.201104691.
[43] A. Das, B. Chakraborty, A.K. Sood, Raman spectroscopy of graphene on different substrates and influence of defects, Bull Mater Sci 31 (2008) 579–584. https://doi.org/10.1007/s12034-008-0090-5.
